# The Paradox of Conspiracy Theory: The Positive Impact of Beliefs in Conspiracy Theories on Preventive Actions and Vaccination Intentions during the COVID-19 Pandemic

**DOI:** 10.3390/ijerph182211825

**Published:** 2021-11-11

**Authors:** Jaesun Wang, Seoyong Kim

**Affiliations:** 1 Department of Public Administration, Division of Global Human Resources, Kangwon National University, Samcheok-si 25913, Korea; jaesunwang@kangwon.ac.kr; 2 Department of Public Administration, Ajou University, Suwon 16499, Korea

**Keywords:** conspiracy theories, belief in conspiracy theories, COVID-19 pandemic, trust, preventive action, vaccination

## Abstract

This study aims to analyze the direct and indirect impact of beliefs in conspiracy theories on COVID-19-related preventive actions and vaccination intentions. The dominant theory in the literature is that beliefs in conspiracy theories have negative consequences. In particular, strong beliefs in conspiracy theories lower people’s intentions to engage in preventive actions and get vaccinated. Previous studies indicated that this dominant theory applies in Korea as well. However, we find that this dominant theory does not apply in Korea. Based on an analysis of survey data, we find that beliefs in conspiracy theories have positive impact on preventive actions and vaccination intentions. In addition, beliefs in conspiracy theories play indirect roles in these two responses to COVID-19. Specifically, when perceived benefits and trust in the government or science enhance preventive actions or increase vaccination intentions, strong beliefs in conspiracy theories promote this effect. This positive role of conspiracy theories is paradoxical because they are generally viewed as negative.

## 1. Introduction

As COVID-19 spreads, a wide variety of conspiracy theories are spreading as well. Lynas [1] lists the following ten conspiracy theories related to COVID-19: “5G is spreading COVID-19”, “Bill Gates is behind the spread of COVID-19”, “the virus escaped from a Chinese lab”, “COVID-19 was created as a biological weapon”, “the US military imported COVID-19 into China”, “COVID-19 doesn’t actually exist”, “the pandemic is being manipulated by the ‘deep state’ of America’s elite”, “COVID-19 is a plot by Big Pharma”, and “COVID death rates are inflated”. These conspiracy theories contradict common sense and have negative connotations about leaders’ use of their power. In addition, these theories focus on the causes rather than the consequences of the COVID-19 pandemic.

The problem is that many people believe in conspiracy theories. Oliver and Wood [2] find, based on nationally representative samples in the US, that over half of the respondents believe a health-related conspiracy theory. According to Mitchell et al. [3], most Americans (71%) have heard of the conspiracy theory that the COVID-19 outbreak was planned, and about one-third of those who are aware of it say that it might be true. Beliefs in concrete conspiracy theories about the pandemic are common in the UK, as 20% of people in the UK agree that “the authorities want us to think that coronavirus is much more dangerous than it really is”, and 19% agree that “the government is deliberately allowing vulnerable people to die” [4]. Moreover, based on a survey of 2254 UK residents aged 16–75 conducted by King’s College London and Ipsos MORI on 20–22 May 2020, Duffy and Allington [5] find that 30% of respondents believe in the conspiracy theories that “coronavirus was probably created in a lab”, “most people in the UK have already had coronavirus without realising it”; and “the COVID-19 death toll is being deliberately reduced or hidden by the authorities”. 

Importantly, beliefs in conspiracy theories negatively impact the fight against COVID-19. Pummerer et al. [6] argue that support for conspiracy theories is likely to jeopardize the success of efforts to fight the pandemic. Romer and Jamieson [7] demonstrate that in the US, beliefs in COVID–19 conspiracy theories are related to a lower perceived threat of the pandemic, which is associated with a lower likelihood of performing preventive actions (e.g., wearing a mask) and of refusing a vaccination even if it becomes available. After reviewing recent research, Douglas [8] concludes that beliefs in conspiracy theories have negative consequences for people’s intentions to comply with government guidelines to reduce the virus spread. In particular, beliefs in conspiracy theories undermine participation in vaccination, which is deeply linked to COVID-19 prevention. Allington et al. [4] report that in the UK, 15% of respondents to a survey believe that “reporters, scientists, and government officials are involved in a conspiracy to cover up important information about coronavirus”. However, 42% of respondents who express that they are unlikely to or will definitely not get vaccinated against the virus agree with this statement.

The reason that conspiracy theories are distributed worldwide is closely linked to fundamental changes in the social structure rather than simply the unique situation of COVID-19. In particular, at the community level, reduced social capital (i.e., a decrease in trust) promotes the development of conspiracy theories. Miller et al. [9] point out that beliefs in conspiracy theories are strong and widespread in Western society because trust in the government is declining. Similarly, Connolly et al. [10] argue that conspiracy theories can be regarded as a marker of institutional distrust. In addition, conspiracy theories are developing because of the increasing role of online media. The development of social networking services based on the Internet is an important driving force for the spread of conspiracy theories. In this respect, Miller et al. [9] explain that this spread is caused by the development of new media. More importantly, conspiracy theories lower trust in institutions and governments, creating a vicious cycle. In the current pandemic, beliefs in COVID-19 conspiracy theories have been shown to decrease support for government regulations and institutional trust [6]. 

Analyzing the role of conspiracy theories in the COVID-19 pandemic is critical for designing public health policies and management in response to the pandemic. This study therefore first discusses the roles of beliefs in conspiracy theories in preventive actions and vaccination from a theoretical perspective. We then analyze whether conspiracy theories directly or indirectly influence COVID-19-related preventive actions and vaccination intentions. In particular, we investigate whether beliefs in conspiracy theories moderate the relationship between the independent variables in the health belief model and the psychometric paradigm and the dependent variables, that is, preventive actions and vaccination intentions. Whereas many previous studies demonstrate that beliefs in conspiracy theories have a negative social influence, we find that beliefs in conspiracy theories increase preventive actions and vaccination intentions. We provide several plausible interpretations of these unexpected findings and present directions for future research.

## 2. Theory and Research Model

### 2.1. Dominant Views about Conspiracy Theories

Research on conspiracy theories has been conducted in various academic fields, and, thus, various definitions of the concept exist. Swami et al. [11] define a conspiracy theory as “a subset of false beliefs in which the ultimate cause of an event is believed to be due to a plot by multiple actors working together with a clear goal in mind, often unlawfully and in secret”. Uscinski et al. [12] argue that a conspiracy theory is a “proposed explanation of events that cites as a main causal factor a small group of persons (the conspirators) acting in secret for their own benefit, against the common good”.

Those definitions are about the same conspiracy theories, but have very different philosophical and fundamental assumptions. In terms of Swami et al. [11], conspiracy theories are prima face irrational beliefs. However, in terms of Uscinski [12], as conspiracies occur, it is not irrational to sometimes engage in conspiracy theorizing. Like Uscinski et al. [12], this study emphasizes the rational aspect of belief in conspiracy theories in that it regards belief in conspiracy theories as rationalized beliefs in a social context under which people construct the knowledge in terms of their own philosophical worldviews. If beliefs about conspiracy theories are not irrational, it can be said that the role of these beliefs is value-neutral and could vary according to changes in situation. Based on this fundamental assumption, this study suggests that belief in conspiracy theories can perform a positive function rather than a traditionally considered negative function.

While conspiracy refers to a real, actual event, conspiracy theory refers to an accusatory perception which may or may not be true [13], p. 3. The conspiracy theories are misunderstood such as conspiracy theories are more popular now, extreme, for the mentally ill, believed by conservative [14]. Moreover, social scientists often view conspiracy theories as misconceptions or incorrect beliefs. However, conspiracy theories are much more than this. Conspiracy theories are tools for dissent used by the weak to balance against power [15]. Therefore, Uscinski [13] argued that conspiracy theories should be treated with skepticism but not as wrong or false per se because conspiracy theories have unique epistemological properties which shield them from falsification. Moreover, Uscinski [13] explained that conspiracy theories played the healthy function to society because they make balance against concentration of power; They accuse an implicitly powerful groups of conspiracy; They gather evidence to challenge the judgment of our prevailing institution [13], p. 5. Democracy requires a check against those in power, a discourse of pros and cons to form a public discourse, and a conspiracy theorist is an important means of checking power and forming public discourse. Moreover, Conspiracy theories can encourage transparency and good behavior by the power. The can foster a healthy skepticism in the public. Conspiracy theories are often used by the weak to balance against power; encourage good behavior and uncover wrongdoing [16], p. 1; take play a crucial role of the market place of ideas and alarm systems [13], p. 6; percolate from the bottom up, and affect policy through direct democracy or by influencing the actions of otherwise nonconspiratorial elites [16], p. 1. When conspiracy theorists are right for the right reasons, they can save the rule of law and idea entrepreneurs [13], p. 6, 9.

It is remarkable that conspiracy theories are neutral concepts, and their judgments are relative such that their function can be changed according to variation in context and culture. Therefore, conspiracy theories should not be treated as wrong [13], p. 4. They have their positive and negative sides. In this vein, most people do not have a consistent rule for accepting some conspiracy theories as true or for rejecting others as false [13], p. 3. In this case, individuals’ beliefs about conspiracy theories are also relative and conditional rather than deterministic. Uscinski et al. [17] argud that there exists a unique predisposition that drives individuals to one degree or another to believe in conspiracy theories. Therefore, the cue suggesting a conspiracy significantly predicted belief in the media conspiracy only among those who did not have strong priors about the conspiracy in the first place—in this case, nonpartisans. Uscinski and Olivella [18] found that the effects of conspiracy thinking on climate change denial are not only larger than previously suggested, but also non-monotonic and conditional on individuals’ party identification.

Previous researchers also investigate why people believe in conspiracy theories. From a personal perspective, conspiracy theories serve as a convenient means or framework for understanding the complex world. According to Douglas et al. [19,20], conspiracy theories help to satisfy individuals’ social-psychological motives, including their epistemic (understanding one’s environment), existential (feeling safe and in control), and social (maintaining positive images of one’s self and group) motives. Thus, individuals believe in conspiracy theories because they have a corresponding motive. Earnshaw et al. [21] argue that beliefs in conspiracy theories give people satisfaction with their environments by making them feel safe.

Conspiracy theories are studied in several academic fields. The differences in the causal factors that determine beliefs in conspiracy theories across different studies reflect differences in the preferences and priorities of each academic discipline’s theoretical backgrounds. Stempel et al. [22] focus on psychological and social structural factors, whereas Douglas et al. [19,20] analyze the roles of political, psychological, and social factors. Kim and Kim [23] set political, social, psychological, and social structural factors as determinants of beliefs in conspiracy theories. Various causal variables intervene in the belief structures for conspiracy theories. For example, Swami et al. [24] show that believing in 9/11 terrorism is related to exposure to conspiracist ideas about 9/11, beliefs in other conspiracy theories, defiance of authority, political cynicism, and the Big Five personality factor of agreeableness. Moreover, De Coninck et al. [25] find that greater exposure to traditional media (i.e., television, radio, and newspapers) is associated with weaker beliefs in conspiracies and misinformation, whereas exposure to politicians, digital media, and personal contacts is associated with stronger beliefs in conspiracies and misinformation.

The social impacts of beliefs in conspiracy theories have both functional and dysfunctional aspects at the individual and social levels. Several theorists have argued that conspiracy theories may have positive outcomes at the individual level. Swami and Coles [26] contend that conspiracy theories may prove useful in understanding individuals’ needs. Beliefs in conspiracy theories promote positive attitudes and motivations toward social issues. For example, Knight [27] and Radnitz and Underwood [28] regard conspiracy theories as the results of people’s attempts to understand social and political realities. Franks et al. [29] describe five types of believers in conspiracy theories and suggest some positive aspects of conspiracy theories, explaining that conspiracy theories can connect with novel aspects. For example, if people hold Type 4 beliefs (i.e., all official narratives are illusions), they have optimism that is conditional on revealing conspiracies. Sasson [30] argues that conspiracy theories may be regarded as motivations for social movements that can create positive change and foster social solidarity. Beliefs in conspiracy theories are linked not only to simple attitudes but also to political actions. For example, these beliefs create opportunities for political debate [31], encourage greater transparency [26], and promote accountability [32]. Moreover, conspiracist beliefs make people work toward collective goals, such as intentions to bring about social change [33,34].

Regarding the functional roles of conspiracy theories at the social level, Sullivan [35] explains that conspiracy theories serve to reveal secrets that elites want to hide and help people understand phenomena that are generally difficult to understand. Moreover, Douglas et al. [20] explain that even if conspiracy theories are secrets aiming to hide those in power, they allow individuals to question the behavior of those in power within the existing dominant hierarchy. Similarly, Clarke [36] reports that conspiracy theories help to reveal actual anomalies in mainstream explanations and allow people to demand greater transparency from governments. Moore [37] regards conspiracy theories as an important component of democratic discourse.

Regarding the dysfunctional roles of conspiracy theories, Bartlett and Miller [38] find that they help to foster political extremism. Douglas [8] suggests that conspiracy theories are associated with political apathy, support for non-normative political actions, climate change denial, vaccine refusal, prejudice, crime, violence, workplace disengagement, and reluctance to adhere to COVID-19 recommendations.

Although discussions of the positive and negative functions of conspiracy theories have been balanced at the theoretical level, empirical studies examining the functions of conspiracy theories focus heavily on their negative effects.

Political science studies identify the negative impacts of beliefs in conspiracy theories on political attitudes and behaviors. In particular, many empirical studies focus on the negative influence of conspiracy theories on trust. For example, individuals reveal that their trust in politics decreased after believing in conspiracy theories [39]. Moreover, conspiracist ideation is significantly associated with anomie, distrust in authority, political cynicism, powerlessness, and self-esteem [40,41]. Pummerer et al. [6] show that believing in conspiracy theories (i.e., explanations for events based on powerholders’ secret arrangements) decreases institutional trust, support for governmental regulations, adoption of physical distancing, and, to some extent, social engagement. Similarly, Imhoff et al. [42] show that beliefs in conspiracy mentalities influence epistemic trust in sources of historical knowledge. A problem with beliefs in conspiracy theories is that they foster prejudice at the social level. Jolley et al. [43] report that people who are exposed to anti-Jewish conspiracy theories exhibit prejudice toward both Jews and people who are not part of the alleged conspiracy. The distrusting attitudes created by beliefs in conspiracy theories negatively affect politics and policy-related behaviors. For example, according to Jolley and Douglas [44], beliefs in conspiracy theories reduce people’s intentions to engage in politics and their carbon footprints. Earnshaw et al. [15] show that strong beliefs in conspiracy theories negatively impact public engagement with pro-health behaviors and public health policies. In addition, people who generally tend to believe conspiracy theories are less likely to register to vote, donate to a political campaign, and display political signs [45]. Moreover, Jolley and Douglas [38] show that people with strong beliefs in conspiracy theories have weaker intentions to increase their energy efficiency and reduce their carbon output. Beliefs in conspiracies lead to illegal actions, such as building occupation [34]. The most negative effect of beliefs in conspiracy theories is that they encourage extremism. Rottweiler and Gill [46] report that conspiracy beliefs are related to violent extremist intentions. Moreover, Bartlett and Miller [38] find that conspiracy theorizing is prevalent in extremist groups online, regardless of whether they hold right- or left-wing ideologies. Conspiracy theories serve as a “radicalizing multiplier” (p. 4) that reinforces ideologies and psychological positions within extremist groups [38].

Whereas many empirical studies focus on the negative functions of conspiracy theories, very few studies demonstrate their positive functions. Miller [31] explains that previous studies on conspiracy theories stress their argumentative role, in which powerful entities are engaged in a grand scheme to control or deceive the masses. He proposes a more positive role of conspiracy theories, that is, the coded social critique role, by which conspiracy theories have an underlying message that critiques various social, political, or economic institutions and actors. These theories can therefore create opportunities for political debate [31]. In addition, Imhoff and Bruder [33] find that conspiracy mentalities play a new role in motivating people’s social actions to change the status quo. Recently, Imhoff and Lamberty [42] find that although hoax conspiracy theories predict resistance to preventive behaviors for COVID-19, beliefs in the conspiracy theory that the virus is a bioweapon are closely associated with self-centered preparatory actions.

### 2.2. Preventive Actions and Conspiracy Theories

Many confirmed cases and deaths have occurred owing to the rapid spread of COVID-19. To prevent catastrophic outcomes, preventive actions at the individual level are socially recommended. Although many people are carrying out these preventive actions, some have provoked public resistance on the grounds that they infringe upon individual liberties.

Many studies have investigated preventive actions against COVID-19, focusing on the factors that determine preventive actions. In particular, these studies analyze the effects of demographic variables on preventive behaviors. For example, Faasse and Newby [46] report that female respondents engage more in health-protective behaviors than their male counterparts do, and younger respondents (ages 18–29) show less engagement in protective behaviors than older respondents do. Studies have focused not only on demographic variables but also on the effects of health- and risk-related variables on preventive behavior. According to Faasse and Newby [46], more exposure to media coverage, heightened concern about an outbreak, higher perceived personal severity of COVID-19, and higher perceived effectiveness of health-protective behaviors significantly increase preventive actions, whereas stronger beliefs in scientific facts about the virus, confidence in government information, and higher COVID-19 knowledge scores decrease preventive actions. Preventive actions are determined by perceived susceptibility [47], perceived severity [48], perceived benefits [49], perceived barriers [49], self-efficacy [50], and cues to action, such as media exposure [48].

Most studies report that beliefs in conspiracy theories have negative effects on preventive behaviors. The negative effects of similar conspiracy theories have been observed in similar previous pandemics as well. For example, Earnshaw et al. [21] show that during the 2014 Ebola epidemic, adherents to conspiracy theories did not support the government’s quarantine policy. The belief that COVID-19 is a hoax is found to be negatively associated with preventive behaviors, such as face mask wearing [7], social distancing [51], and hand washing and maintaining physical distance [36]. Beliefs in conspiracy theories affect preventive actions not only at the individual level but also at the public level. Allington and Dhavan [52] find a negative relationship between beliefs in COVID-19 conspiracy theories and compliance with public health recommendations. Freeman et al. [53] argue that beliefs in coronavirus conspiracies are related to lower adherence to all government guidelines. Such beliefs are also found to be negatively related to compliance with social distancing guidelines [54,55].

These studies have the limitation that they focus on the direct influence on preventive actions and ignore the indirect role of conspiracy theories. However, conspiracy theories exert both direct and indirect influences through mediation and control. For example, Biddlestone et al. [54] show that people with individualist (as opposed to collectivist) values are less likely to engage in COVID–19 preventive behaviors, and they find that this relationship is mediated by beliefs in COVID–19 conspiracy theories. In addition, Banai et al. [56] use a multiple mediation analysis to show that conspiracy beliefs are indirectly associated with compliance via trust in government officials. Conversely, some studies contradict the dominant finding that conspiracy beliefs have negative effects, finding no correlation between beliefs in COVID-19 conspiracies and recommended behaviors to prevent COVID-19 [57,58].

### 2.3. Vaccination and Conpiracy Theories

**Figure 1:**
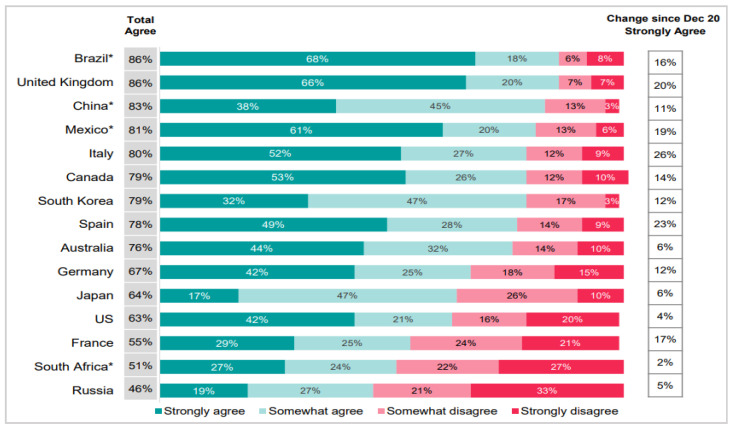
Cross-country Variation in Vaccination Intentions. (Source: Ipsos Global Advisor 14–17 January 2021. Reprinted with permission from Page (2021) [60]. 2021©Ipsos Global Advisor.) Note: Respondents were asked their level of agreement with the statement “If a vaccine for COVID-19 was available, I would get it”. (n = 12,777 online adults aged 16–74 across 15 countries (excluding those who reported having received the vaccine)). * Online respondents in Brazil, China, Mexico, Russia, and South Africa tend to be more urban, educated, and affluent than the general population.

Vaccination is an effective intervention for controlling infectious diseases. It is regarded as a routine, effective measure for controlling the spread of COVID-19 [59]. Because of the high risks associated with the COVID-19 pandemic, many presume that intentions to be vaccinated or actual vaccination rates are high, but this idea is not necessarily true in practice. As a result, vaccination intentions and rates vary considerably across countries, as Figure 1. According to one survey, 86% of respondents in Brazil but only 46% of respondents in Russia expressed agreement with the statement “If a vaccine for COVID-19 were available, I would get it”.

Several studies show that intentions to receive the COVID-19 vaccine are very high. Based on a meta-analysis, Wang et al. [61] find that studies using representative samples report a willingness rate of 73.16%. Similarly, other meta-analyses find that the proportion of respondents willing to receive a COVID-19 vaccination is 68.4% [61,62,63]. However, it is noteworthy that 20–30% of people do not report intentions to vaccinate. For example, Jone [64] reports that one in five adults in the US (18%) can be regarded as vaccine-resistant. These responses are consistent over time. When asked “if one of the FDA-approved vaccines to prevent coronavirus/COVID-19 was available to you right now at no cost, would you agree to be vaccinated”, 19%, 20%, and 18% of respondents chose “would not agree to be vaccinated” and “not likely to change mind” for surveys run from 18–23 May 2021, 14–20 June 2021, and 19–26 July 2021, respectively.

Because some people express reluctance toward being vaccinated against COVID-19, empirical studies have investigated the factors that affect vaccination intentions. Demographic variables appear to be systematically related to these intentions. Based on a meta-analysis of 39 studies related to vaccination, Wang et al. [61] report that women are less likely to accept the vaccine than men are (odd ratio (OR): 0.728, 95% confidence interval (CI): 0.613, 0.865). In addition, compared to respondents with a high school education (equivalent) or below, those with a college degree or higher exhibit greater intentions to receive the COVID-19 vaccine (OR: 1.613, 95% CI: 1.212, 2.145). In addition, Lazarus et al. [65] show that higher household income is a positive predictor of willingness to be vaccinated against COVID-19. Rieter et al. [66] show that people with higher education levels, greater household incomes, and liberal ideologies express more willingness to be vaccinated. Detoc et al. [67] show that men, older people, and healthcare workers have higher COVID-19 vaccine acceptance. In addition, Mahmud et al. [68] show that older people, healthcare workers and professionals, and people who received the flu vaccine are more likely to have positive intentions toward vaccination.

In addition to considering demographic variables, studies analyze the influences of individual health factors or risk perception factors on vaccination intentions. For example, Wang et al. [61] report that the perceived susceptibility and severity of COVID-19 and the perceived benefits and risks of acceptance affect people’s willingness to vaccinate. Several studies find a positive relationship between perceived COVID-19 infection risk and willingness to receive a COVID-19 vaccine [46,67]. Similarly, Detoc et al. [67] show that greater fear of COVID-19 and higher perceived individual risk increase COVID-19 vaccine acceptance. Recent studies find that various other variables influence vaccination intentions. For example, Rieter et al. [66] show that knowledge, perceived severity, the perceived stigma of a COVID-19 infection, the perceived effectiveness against potential harm, the perceived unavailability of COVID-19 vaccines, self-efficacy, and perceived positive social norms regarding protective behaviors against COVID-19 have positive impacts on COVID-19 vaccine acceptance. In addition, based on a population survey in Hong Kong, Wong et al. [69] show that perceived severity, perceived benefits of the vaccine, cues to action, self-reported health outcomes, and trust in the healthcare system or vaccine manufacturers are positively associated with COVID-19 vaccination, whereas perceived barriers to access and perceived harm are negatively correlated with vaccination.

Vaccination actions and intentions are related to beliefs in conspiracy theories. In the UK, conspiracies regarding the COVID-19 pandemic are widespread among vaccine-hesitant individuals. Allington et al. [4] report that 27% of the UK public agree that “the real truth about coronavirus is being kept from the public”. However, this proportion increases to 64% among people who say that they are unlikely to or definitely will not be vaccinated. In addition, 51% of the vaccine-hesitant believe that “an impartial, independent investigation of coronavirus would show once and for all that we’ve been lied to on a massive scale”, whereas 21% of those who do not believe conspiracy theories agree with that statement. Jolley and Douglas [70] show that those who believe anti-vaccine conspiracy theories have lower vaccination intentions. Finally, Craciun and Baban [71] conduct qualitative research on people’s vaccination decisions and find that conspiracy theories play a significant role in these decisions.

A limitation of the above-mentioned studies is that they focus on the direct negative influences of beliefs in conspiracy theories on vaccination intentions. The positive impacts and indirect effects of beliefs in conspiracy theories on vaccination intentions have been less examined in prior studies.

### 2.4. Research Model

The above-mentioned studies focus on the direct positive or negative effects of beliefs in conspiracy theories. In contrast, this study focuses on both direct and indirect effects of these beliefs. This study investigates the moderating roles of beliefs in conspiracy theories in the relationships between preventive actions and vaccination intentions and their antecedents. Few studies examine the moderating effects of conspiracy theories. One such study, by Gu et al. [72], finds a moderating rule of beliefs in conspiracy theories; in states with more online attention to COVID-19 conspiracy theories, the negative effects of attention to conspiracy theories are much weaker than in states with less concern about conspiracies.

**Figure 2:**
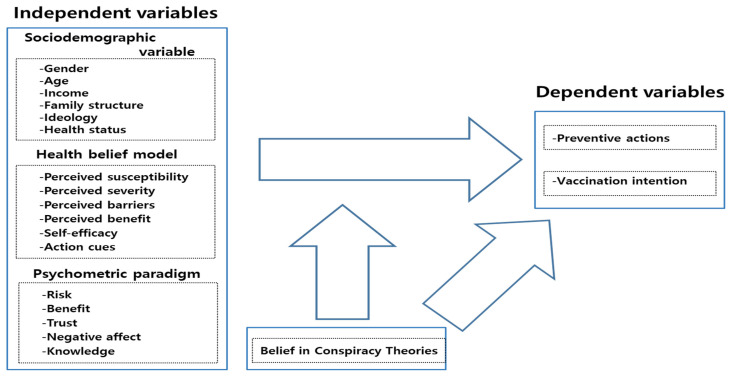
Research Model.

Figure 2 shows the research model used in this study. We set preventive actions and vaccination intentions related to COVID-19 as the dependent variables. We set six variables suggested by the health belief model and five variables emphasized by the psychometric paradigm model as independent variables. Based on the health belief model, Kim and Kim [23] show that self-efficacy and perceived severity positively impact preventive actions. In addition, preventive actions are determined by perceived susceptibility [49], perceived benefits [49], perceived barriers [49], and cues to action [48]. Similarly, Chen et al. [73] show that perceived susceptibility and self-efficacy in preventing COVID-19 contribute to engagement in preventive behaviors. Mahmud et al. [68] demonstrate that among health belief models, perceived susceptibility to and severity of COVID-19 (*p* < 0.001) and perceived benefits from the vaccine (*p* < 0.001) are positively associated with vaccination intentions, whereas perceived barriers are negatively associated (*p* < 0.001) with these intentions. Individuals are more likely to receive the vaccine after obtaining complete information (*p* < 0.001) and when vaccine uptake is more common among the public (*p* < 0.001). Moreover, based on the health belief model, Wong et al. [64] show that perceived severity, perceived benefits of the vaccine, cues to action, self-reported health outcomes, and trust in the healthcare system or vaccine manufacturers are positively correlated with COVID-19 vaccine acceptance, whereas perceived barriers to access and perceived harm are negatively correlated with vaccination. Handebo et al. [74] find that perceived susceptibility, perceived benefits, perceived barriers, and cues to action are significantly associated with intentions to receive the COVID-19 vaccine.

In contrast, according to the psychometric paradigm [75], risk and benefit perceptions, trust, negative affect, and knowledge impact preventive actions and vaccination intentions. Perceived risks and benefits are key influencers of preventive actions. For example, Shahin and Hussien [76] find a significant positive correlation between the perception of the seriousness of COVID-19 and self-efficacy in handling COVID-19. This result implies that perceptions of COVID-19 risks may promote preventive actions. Perceived benefits and knowledge are significantly associated with preventive behaviors [73]. Trust in the government is positively associated with officially recommended preventive behaviors. However, this role of trust is mediated by knowledge; Min et al. [77] report that a positive relationship between trust and excessive preventive behaviors appears only among those with low levels of COVID-19 knowledge. Both the rational and emotional aspects of risk influence preventive actions. For example, preventive actions, such as promoting hygiene and cleaning, are influenced by negative attitudes toward the coronavirus and are mediated by an affective appraisal of risk [78]. In addition, emotional responses to food safety incidents significantly increase risk perception and prevention actions [79].

People’s intentions to get vaccinated are also influenced by risk perception, which, in turn, is influenced by affect [80]. In addition, perceived risks (−) and benefits (+), knowledge (+), and social trust (+) significantly impact vaccination intentions [81].

## 3. Sample and Measures

We collected survey data (*N* = 1525) from a sample of people in Korea from 6 August 2020, to 11 August 2020. Korea Research, a survey research institute, executed the survey online using an online panel and a web survey system. Korea Research’s online panel contains 460,000 candidate survey respondents, and an e-mail with the web address for the survey questions was sent to 9839 of them. These e-mails were opened by 2083 people, and 1525 of them answered all of the questions (see Table 1). To secure a representative sample of the Korean population, we used a quota sampling method such that the proportions of respondents by region, gender, and age reflect the general population. With random sampling, the survey has a sampling error of ±2.5% at the 95% confidence level.

Questions about pre1ventive behaviors referred to recommendations by the government, the World Health Organization, and other healthcare organizations. We measure preventive actions in response to the COVID-19 pandemic using 19 items, including wearing a mask, covering one’s mouth with one’s sleeve when coughing, and washing hands for at least 30 s. The answers are scored on a five-point scale (1 = do not comply at all, 2 = slightly do not comply, 3 = moderately comply, 4 = somewhat comply, 5 = highly comply). Vaccination intentions are measured on a five-point scale according to respondents’ levels of agreement with two statements. Beliefs in conspiracy theories are measured using seven items chosen based on previous studies of conspiracy theories [82,83]. The seven questions related to these items are structured to include politicians, governments, countries, and pharmaceutical companies, which are the subjects of relevant conspiracies [23]. Greater agreement with these conspiracy theories implies stronger beliefs in conspiracy theories.

When compositing multiple measurement items into one variable, a simple average of multiple items was used Except for action cues and trust in experts, most of the responses for each question are measured on a five-point Likert scale. Table 2 describes the content and shows the reliability scores (Cronbach’s α) of the measurement items for each variable.

## 4. Analysis and Findings

### 4.1. Descriptive Analysis

To analyze key variables measured in the survey, such as preventive actions, vaccination intentions, and beliefs in conspiracy theories, we derive the average values for each major demographic variable and conduct analysis of variance (ANOVA) tests. The results are shown in Table 3.

First, with regard to preventive actions, women, older people, people with higher incomes, more educated people, people with more young children or elderly family members, more ideologically progressive people, and people whose health deteriorated after COVID- 19 take more preventive actions. Among these, the differences between genders, age groups, and families with different numbers of elderly people are statistically significant.

Next, vaccination intentions are greater for female respondents, those in their 60s or older, those with incomes of five million won or more, those with low education levels, those with large numbers of children or elderly family members, ideologically conservative respondents, and those whose health deteriorated after COVID-19. Among them, age, education level, the number of elderly family members, and health after COVID-19 have significant effects. The negative role of education in this case contradicts a previous finding that the intention to receive a COVID-19 vaccine is explained by having a bachelor’s degree [74].

Lastly, beliefs in conspiracy theories are stronger among women, younger people, people with lower incomes, people with less education, people with more children and elderly family members, more ideologically conservative people, and people in worse health. Among these variables, household income, the number of children, ideology, and health deterioration have significant effects.

The dominant result of previous studies is that strong beliefs in conspiracy theories can reduce preventive behaviors [54,55,56,57,58,59,60] and vaccination intentions [4,69]. This logic holds in this study in the case of age and income. Lower ages and incomes are associated with stronger beliefs in conspiracy theories, less preventive behavior, and lower vaccination intentions. It is noteworthy that people whose health deteriorated after COVID-19 exhibit stronger beliefs in conspiracy theories and greater vaccination intentions. These results suggest that conspiracy theories may play a role in inducing vaccination when health is deteriorating, indicating that beliefs in conspiracy theories may not necessarily play a negative role.

### 4.2. Correlation Analysis

We perform simple and partial correlation analyses to analyze the relationships among the variables. In the partial correlation analysis, the control variables are gender, age, household income, education level, number of children, number of elderly people, ideology, and health change after COVID-19. In Table 4, the numbers below the diagonal line are the results of the simple correlation analysis, and those above the diagonal line are the results of the partial correlation analysis.

First, we investigate the relationships among preventive actions, vaccination intentions, and beliefs in conspiracy theories, which play a key role in this study. We find a statistically significant positive correlation between preventive actions and vaccination intentions, which results from the fact that the two actions play similar roles in the response to COVID-19. However, because their correlation coefficient is not large, the two variables may have some degree of independence. This result suggests that those who take preventive actions may not necessarily intend to be vaccinated. Beliefs in conspiracy theories are positively related to vaccination intentions but are not significantly correlated with preventive actions. This relationship contradicts the general findings that strong beliefs in conspiracy theories decrease preventive actions and vaccination intentions.

Preventive actions are positively related to perceived severity, perceived benefits, self-efficacy, media exposure, risk perception, benefit perception, trust in the government, trust in experts, trust in science, negative affect, knowledge, perceived susceptibility, and perceived barriers. From a logical perspective, preventive actions should increase when perceived susceptibility increases, but we observe the opposite relationships.

In terms of the magnitudes of the correlation coefficients, preventive actions have the largest correlation with self-efficacy, followed, in order, by benefit perception, risk perception, knowledge, perceived severity, and trust in the government. Both health belief factors and the psychometric paradigm influence preventive actions. Vaccination intentions are positively related to perceived susceptibility, perceived severity, perceived barriers, media exposure, risk perception, trust in experts, trust in science, and knowledge and are negatively related to self-efficacy. When self-efficacy is high, vaccination intentions decrease. This result may arise because self-efficacy includes a sense of control, which is closely associated with confidence in one’s ability to control COVID-19. This confidence paradoxically lowers vaccination intentions. Vaccination intentions are most correlated with perceived barriers and perceived susceptibility, followed, in order, by risk perception, knowledge, trust in science, trust in experts, and perceived severity.

Beliefs in conspiracy theories are positively related to perceived susceptibility, perceived barriers, media exposure, and risk perception and are negatively related to perceived benefits, self-efficacy, benefit perception, trust in the government, and knowledge. Factors such as benefits, trust, and knowledge diminish beliefs in conspiracy theories, whereas risk-related factors increase beliefs in conspiracy theories. Beliefs in conspiracy theories have the largest correlation with trust in the government, followed, in order, by perceived barriers, self-efficacy, benefit perception, perceived susceptibility, perceived benefits, and risk perception.

Interestingly, the variables with the highest correlations with the three key variables differ. Self-efficacy, perceived barriers and perceived susceptibility, and trust in the government have the highest correlations with preventive actions, vaccination intentions, and beliefs in conspiracy theories, respectively. These results suggest that the influencing factors of each behavior may differ.

### 4.3. Regression Analyses

We first perform a regression analysis with preventive actions as the dependent variable and the four factors as independent variables. Table 5 shows that among the demographic variables, the degree of preventive action decreases as age, education, the number of elderly people in the household, and deterioration in health status after COVID-19 increase. Before performing this analysis, we checked the preconditions for regression analysis. Multicollinearity is not found because the tolerance is greater than or equal to 0.1 and VIF is less than 10 for all variables. The Durbin-Watson value is 1.974 in preventive action and 1.847 in vaccination, indicating that there is no residual independence problem between the reference values 1 and 3. To verify the model, stepwise was performed, please see Model 1, 2, 3, 4 in Table 5 and Table 6.

Based on Model 4, Based on the standardized beta values, women take more preventive actions. This result is interpreted as reflecting women’s sensitivity to risk.

Among the health belief factors, compliance with preventive behavior increases when perceived susceptibility, self-efficacy, and exposure to media increase, and it decreases when perceived barriers increase. Susceptibility and barriers increase vulnerability to COVID-19. Thus, preventive actions are higher when their costs and barriers are lower. The increase in preventive behavior when self-efficacy increases may be due to the fact that preventive actions are a means of suppressing the COVID-19 pandemic, which is linked to a sense of control, an intrinsic element of efficacy. The effect of high exposure to the media suggests that many messages are being delivered through the media, which increases people’s perception of risk in the context of the COVID-19 pandemic. Self-efficacy has the largest standardized beta value. This result suggests that perceptions of control play an important role in preventive actions.

Among the psychometric paradigm variables, perceived risk, perceived benefit, trust in the government, trust in experts, and knowledge induce preventive actions. People take more preventive actions when the risk of COVID-19 impacts them more and when the likelihood of solving the COVID-19 problem increases. Trust in the government and trust in experts are key factors in implementing quarantines and COVID-19 prevention. In addition, preventive actions increase as knowledge about COVID-19 increases. Based on the standardized regression coefficients, knowledge has the greatest explanatory power, followed by perceived benefits and risks. These results suggest that the diffusion of related knowledge and education is important for inducing preventive actions.

Finally, beliefs in conspiracy theories are found to induce preventive actions. This finding is contrary to previous results about conspiracy theories, which suggest that beliefs in conspiracy theories may reduce preventive behavior. We provide an interpretation of these results in the discussion section.

The explanatory power of the full model is 32%. The psychometric factors have the greatest explanatory power of the three factors. These results suggest that the risk perception aspects rather than the health aspects of COVID-19 play an important role in preventive actions.

Table 6 shows the results of the regression analysis with vaccination intentions as the dependent variable. First, the results for the demographic variables indicate that vaccination intentions increase if there are more children in the household or if a person’s health deteriorates after COVID-19. However, vaccination intentions decrease as the level of education increases. This result is unusual. We argue that less educated people are more likely to be vaccinated because they face a greater threat from COVID-19 and lack the resources to defend against it. In such a high-risk, low-resource situation, those with lower education levels are likely to depend more on vaccines.

Among the health belief factors, perceived susceptibility and perceived barriers are positively related to vaccination intentions, whereas vaccination intentions decrease when self-efficacy increases. We also find the unusual result that vaccination intentions are lower when people face many obstacles to health actions. This result may occur because the components of the obstacles are mainly focused on preventive actions, and, thus, their direct relationship with vaccination is not strong. When a person faces many obstacles to preventive behavior, they are highly likely to avoid obstacles through vaccination. A reason that vaccination intentions decrease when self-efficacy increases may be that if people consider the side effects of vaccines as a loss of control, they will feel a conflicting sense of their own control, which is inherent in self-efficacy.

In the psychometric paradigm, risk perception, trust in the government, trust in experts, trust in science, and knowledge have positive effects on vaccination intentions. Based on the standardized regression coefficients, the variable with the greatest influence on vaccination intentions is risk perception, followed by trust in science and technology and knowledge. The fear of COVID-19 might depend on reason-based science and technology. Finally, beliefs in conspiracy theories increase vaccination intentions. These results contradict the results of previous studies [4,70].

Based on the R^2^ value, the overall model’s explanatory power is 19.9%. Because the explanatory power is not high, it is necessary to find additional variables that can explain vaccination intentions. The health belief and psychological perception factors have equivalent explanatory power. When comparing the determinants of preventive actions and vaccination intentions, the variables affecting just preventive actions are gender, age, number of elderly family members, exposure to media, and number of children, and the factors that influence both dependent variables are the level of education, deterioration in health after COVID-19, and trust in science. In addition, perceived susceptibility, perceived barriers, self-efficacy, risk perception, benefit perception, trust in the government, trust in experts, knowledge, and beliefs in conspiracy theories commonly influence the two dependent variables in the same direction. Interestingly, education level, perceived barriers, and self-efficacy play opposite roles in preventive actions and vaccination intentions.

### 4.4. Moderation Analysis

This study analyzes both the direct and indirect effects of conspiracy theories. To examine the indirect effects, we analyze the moderating effect of beliefs in conspiracy theories. This analysis follows the methods and procedures suggested by Barron and Kenny [84]. We first perform statistical verification on the 28 interactions between beliefs in conspiracy theories, the health belief factors, and the psychometric paradigm factors. Among them, only four interaction terms are found to be significant. Appendix A provides details of these four significant terms.

**Figure 3:**
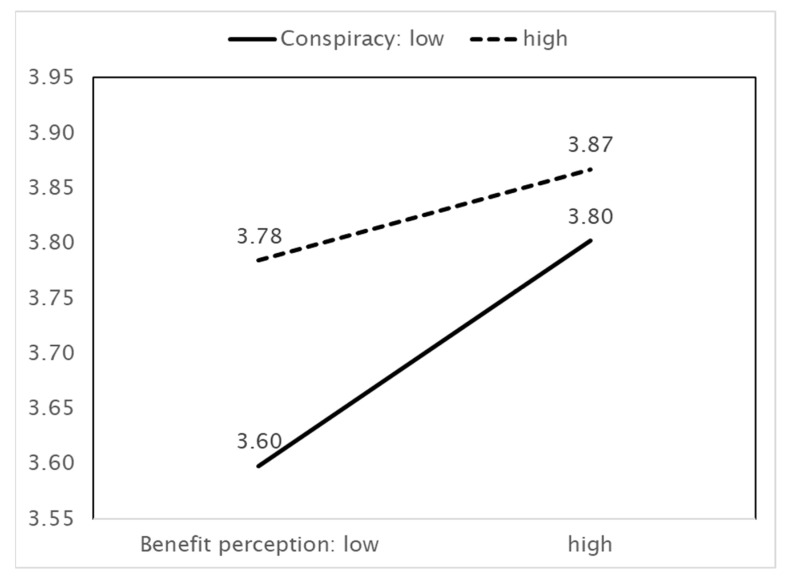
IV (Benefit perception) × MV (Belief in conspiracy theories) = DV (Preventive actions).

**Figure 4:**
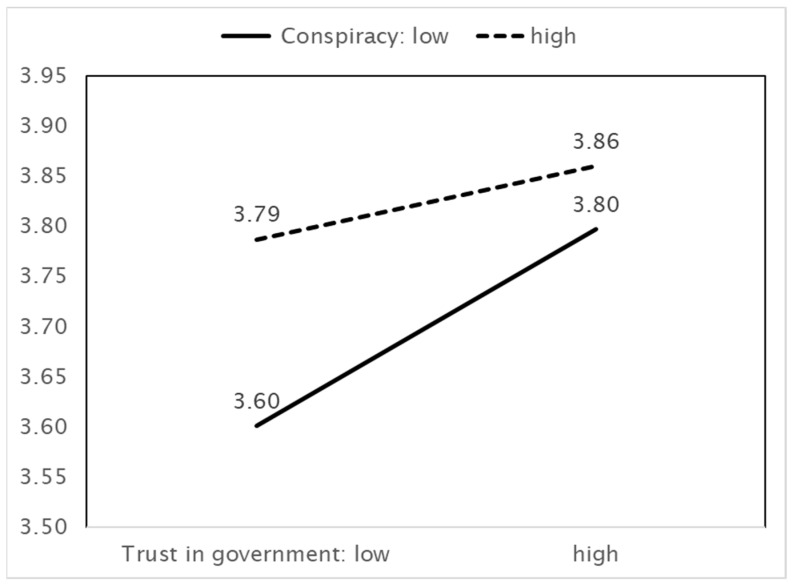
IV (Trust in government) × MV (Belief in conspiracy theories) = DV (Preventive actions).

As Figure 3 shows, higher benefit perceptions are associated with more preventive actions. In this case, the effect depends on beliefs in conspiracy theories; when these beliefs are greater, benefit perception has a positive effect on preventive actions. Conspiracy theories therefore play a role in facilitating preventive actions. However, this effect is weaker when the perception of benefits increases. Next, Figure 4 shows that the effect of trust in the government on preventive actions also depends on beliefs in conspiracy theories. Stronger beliefs in conspiracy theories facilitate the increasing effect of trust in the government on preventive actions. This effect weakens as trust in the government increases.

**Figure 5:**
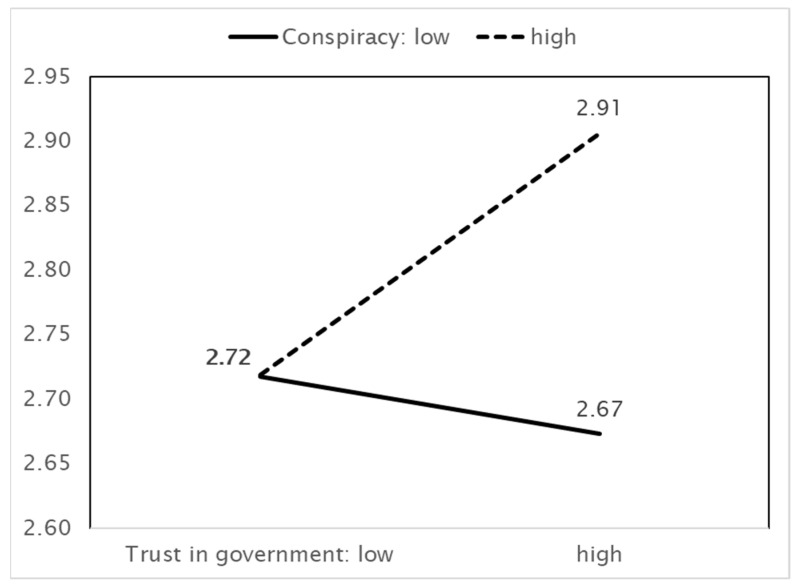
IV (Trust in government) × MV (Belief in conspiracy theories) = DV (Vaccination intentions).

In Figure 5, the effect of trust in the government on vaccination intentions depends on beliefs in conspiracy theories. Conspiracy theories do not play a role when trust in the government is low, but they play the opposite role when trust in the government is high. That is, when trust in the government increases, strong beliefs in conspiracy theories increase vaccination intentions, whereas weak beliefs in conspiracy theories reduce vaccination intentions. Consequently, strong beliefs about conspiracy theories are the driving force behind vaccination intentions. When trust is low, beliefs about conspiracy theories don’t work because both trust and belief in conspiracy theories share a negative orientation. On the other hand, among those who have high trust in the government, strong belief in conspiracy theories increase positive feelings toward vaccination intention because they need the means to survive in the wicked world fulling with conspiracy plots.

**Figure 6:**
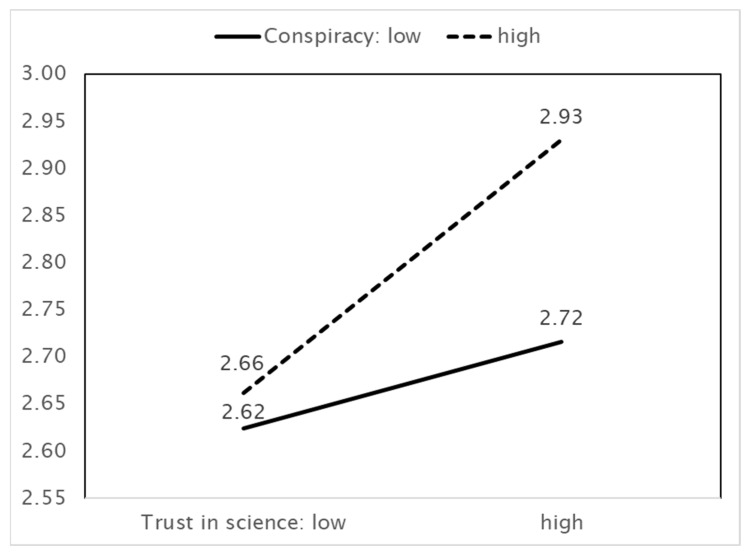
IV (Trust in science) × MV (Belief in conspiracy theories) = DV (Vaccination intentions).

In Figure 6, greater trust in science implies greater vaccination intentions. When trust in science increases, vaccination intentions increase, and this effect arises when beliefs in conspiracy theories are strong rather than when they are weak.

## 5. Main Findings and Discussion

The purpose of this study was to analyze the direct and indirect effects of conspiracy theories on COVID-19 preventive behaviors and vaccination intentions. The results of the empirical analysis based on the survey data can be summarized as follows.

First, the dominant finding of previous studies of conspiracy theories is that these theories reduce preventive actions and vaccination intentions. However, our study found that beliefs in conspiracy theories have increased COVID-19 preventive actions and vaccination intentions. Using a regression analysis, we found that beliefs in conspiracy theories directly increase preventive actions and vaccination intentions. We also verified the indirect effects of beliefs in conspiracy theories by analyzing their moderating effects. Benefit perception and trust in the government enhance preventive actions, but this effect is promoted by strong beliefs in conspiracy theories. In addition, trust in the central government and trust in science increase vaccination intentions, but these relationships strengthen when the effect of conspiracy theories is strong.

These results suggest that beliefs in conspiracy theories increase preventive actions and vaccination intentions during the COVID-19 pandemic. However, very few studies find positive effects of beliefs in conspiracy theories. Several hypothetical explanations for our results are as follows.

First, this study showed that belief in conspiracy theories is performing a positive function. However, when interpreting these results, it is necessary to consider various other possibilities without excluding them. Since the data of this study were collected in Korea, the findings might reflect the particularity of Koreans. Because Korea has a strong collectivist culture, people tend to have positive orientation for the government’s preventive actions and vaccinations. Due to this cultural value, strong belief in conspiracy theories can appear to have a close positive relationship with the two behaviors. These results suggest that the positive function of conspiracy theories may not appear in countries with different cultural contexts. Our paradoxical findings may come from cultural effect. Butter and Knight [85] criticized that existing conspiracy theory studies have a problem in that they reflect the value bias of the US or the West. Unlike the West, in Eastern collectivist culture, belief in conspiracy theory has the possibility to do positive functions.

In the collective Eastern culture, the public welling is stressed so that not only my health but also the health of others is important, but in the individual Western culture, vaccination and health behavior are depending on individual wills and their choice. Such cultural differences brought about the difference in preference and behavior between the East and the West. Moreover, they linked to the beliefs in conspiracy theories. Recently a few empirical research has been tested this assumption. For example, Biddlestone et al. [54] demonstrated that individualism negatively increased the engagement in social distancing whereas collectivism, which closely connected with higher belief in conspiracy theories, positively predicted both social distancing and hygiene-related intentions. Also, after investigating the links between cultural values and belief in conspiracy theories, Adam-Troian et al. [86] reported that positive associations between masculinity, collectivism, and CT beliefs.

In short, we need to make sure that we are not talking about conspiracy theories generally in this study. However, we may look at the particular conspiratorial claims which are doing the work in Korea or any people.

Another possibility is about the measurement item used to examine the beliefs in conspiracy theories. Generally, measures for conspiracy theories may hold the negative content with which most people cannot agree with them. In the other, if those measurements did not contain the negative connotation, they may fail to measure the belief in conspiracy theory. This study adopted the measures that describe what is generally happening due to distrust in modern society (e.g., ‘politicians do not honestly reveal their true intentions to the public regarding their decisions on coronavirus disease (COVID-19)’, ‘the government is always monitoring the public’); that related to weak conspiracy theories rather than strong conspiracy theories (e.g., ‘there is a secret organization that greatly influences political decisions’, ‘the government is hiding something from the public’, ‘the government makes important decisions related to coronavirus disease (COVID-19) without the public knowing’); that representing the widespread distrust toward companies (e.g., ‘coronavirus disease (COVID-19) was deliberately created by pharmaceutical companies to make money’); That are publicly discussed (e.g., certain powerful nations deliberately created the coronavirus (COVID-19) to dominate the world). Such generality, weakness, widely accepted, publicly discussed attributes in measure usher into more positive reaction. Conversely, if we designed measurement items in conspiracy theories with more strong distrust, strong secrecy, widely accepted, not publicly discussed, there will be different results from present studies. Even though conspiracy theories are prevalent in Korea, if the measurement that can reveal them were not properly measured, there was high possibility of errors in findings and reinterpretation in the current studies. This requires further elaborate study on the measurement of belief in conspiracy theories in terms of Korea.

A third possibility is that, as the survey results show, in Korea, very few people believe in conspiracy theories. If only a few people believe, there is very little variation in the overall response. Such little variation can have a sensitive effect on the results, i.e., the positive or negative function of belief in conspiracy theory.

Fourth, the function of beliefs in conspiracy theories may vary depending on the context and circumstances. Conspiracy theories may play a different role during pandemics than they do in normal situations. In normal times, conspiracy theories have negative impacts on specific actions, but in emergencies, conspiracy theories may have positive functions. For example, in a crisis situation, conspiracy theories can promote social skepticism, which is linked with proactive behavior.

Fifth, strong beliefs in conspiracy theories are likely to cause reactions. In some, less common cases, stronger negative thinking may induce positive rather than negative behaviors. Pessimistic thinking can trigger positive behaviors. For example, pessimistic beliefs about climate change induce people to take active measures to prevent it.

Sixth, this finding may be the result of bias in the measurement items. We analyzed the correlation between preventive actions/vaccination intentions and seven measure for belief in conspiracy theories individually (please, see Appendix B). There is little correlation between preventive action and each of the seven item measuring belief in conspiracy theories. Even if there was a correlation, the results are inconsistent. These results are interpreted to have influenced the belief in conspiracy theories to increase preventive behavior. Moreover, the items measuring conspiracy theories include whether people believe conspiracy theories themselves and whether they believe various stories that constitute a specific conspiracy theory. In the latter case, the directions of conspiracy theories’ influence may vary depending on their contents. Different stories about conspiracy theories may result in different responses. Finally, the current association may be a peripheral relationship. There may be third variables that induce preventive behavior and vaccination intention.

These interpretations are necessarily provisional and require further verification. The fact that conspiracy theories can perform positive functions has new implications for conspiracy theory management. At a practical level, if conspiracy theories have positive functions, it is necessary to actively utilize them rather than eliminating them. At the theoretical level, deliberate discussions of conspiracy theories’ functional roles are necessary. In addition, it is necessary to develop a scale for measuring the positive and negative functions of conspiracy theories and verify the roles of both functions in practice.

## 6. Conclusions and Limitations

This study empirically analyzed the direct and indirect effects of beliefs in conspiracy theories. Beliefs in conspiracy theories play a direct role in increasing preventive actions and vaccination intentions for COVID-19, and they indirectly moderate the effects of perceived benefits, trust in the government, and science on preventive actions and vaccination intentions. The findings differ greatly from the dominant understanding that conspiracy theories play mainly negative roles. Logically, the result that conspiracy theories lead to productive and positive outcomes is paradoxical because they can be attributed to distrust. A new theoretical approach is needed to explain this paradoxical role of conspiracy theories. This study aims to serve as a starting point for such research in the future.

This study has several limitations. First, the findings may have limited generalizability because they were measured in the context of Korea. Second, because the measurement items of conspiracy theories were adopted for this study and were not used in the prior studies, follow-up analyses on the generalizability of the measurement scale are needed. Third, because the result that conspiracy theories performs positive functions is very unique, the theoretical basis for it is rather weak. These limitations should be addressed in future studies.

## Figures and Tables

**Table 1:** Distribution of survey respondents.

Categories		N	%	Categories		N	%
All respondents		1525	100	Education level	High school	720	47.2
Gender	Men	731	47.9		College	805	52.8
	Women	794	52.1	No. of children	0	1085	71.1
Age	18–29	254	16.7		1	241	15.8
	30–39	248	16.3		2+	199	13.0
	40–49	299	19.6	No. of elderly people	0	859	56.4
	50–59	310	20.3		1	279	18.3
	60+	414	27.1		2+	386	25.3
Household income	<299 MW	499	32.7	Ideology	Conservative	714	46.8
	300–499 MW	577	37.8		Progressive	811	53.2
	>500 MW	449	29.4	Health status change after COVID-19	Not worse	668	43.8
					Worse	857	56.2

**Table 2:** Variable measurement and reliability.

Factor	Variable	Measures	Reliability
Preventive actions		(1) wearing a mask; (2) covering one’s mouth with one’s sleeve when coughing; (3) washing one’s hands for at least 30 s; (4) refraining from traveling or going out; (5) ventilating rooms at least twice a day; (6) social distancing; (7) staying at home for three to four days if sick; (8) not going where there are many people; (9) using hand sanitizer to clean one’s hands; (10) refraining from visiting hospitals; (11) avoiding visiting public places; (12) not holding meetings with people; (13) keeping a distance of two arms’ length from people; (14) refraining from using public transportation; (15) staying two meters away from people in daily life; (16) eating health foods, such as vitamins; (17) periodically disinfecting things that one touches; (18) avoiding touching one’s eyes, nose, or mouth with one’s hands; (19) disinfecting cell phones	0.926
Vaccination intentions		- If the COVID-19 vaccine is available, I will apply for vaccination first. - Even if there are side effects, I plan to use the COVID-19 vaccine early.	0.649
Beliefs in conspiracy theories		- Politicians do not honestly reveal their true intentions to the public regarding their decisions on coronavirus disease (COVID-19). - There is a secret organization that greatly influences political decisions. - The government is hiding something from the public. - The government is always monitoring the public. - The government makes important decisions related to coronavirus disease (COVID-19) without the public knowing. - Certain powerful nations deliberately created the coronavirus (COVID-19) to dominate the world. - Coronavirus disease (COVID-19) was deliberately created by pharmaceutical companies to make money.	0.852
Health belief factors	Perceived susceptibility	- I am more likely to be at risk for COVID-19 than others are. - I live in an environment where I can be exposed to COVID-19 infection.	0.759
	Perceived severity	- Diseases caused by COVID-19 infection have very serious consequences. - Diseases caused by COVID-19 infection will have a major impact on my life.	0.781
	Perceived barriers	- Excessive efforts are necessary to comply with actions for COVID-19 prevention. - There are many obstacles to complying with actions for COVID-19 prevention.	0.503
	Perceived benefit	- The benefits of complying with actions for COVID-19 prevention outweigh the costs. - The benefits of taking actions for COVID-19 prevention outweigh the inconvenience.	0.575
	Self-efficacy	- If I try, I can fully practice preventive actions. - I am sufficiently able to take actions for COVID-19 prevention.	0.865
	Action cues 1: Exposure to media	How much COVID-19-related information do you obtain from the following sources: - offline media (broadcasting, paper newspapers, magazines, etc.) - online media (Internet newspapers, portal news, etc.) - Internet sources (personal blogs, social networks, cafes, and communities). → Response scale: (1) I did not get information at all; (2) I did not get much information; (3) I got information; (4) I got some information, (5) I got a lot of information.	0.603
	Action cues 2: Knowing confirmed cases	-Has anyone you know had a confirmed case of coronavirus? → Response scale: (1) No; (2) Yes.	-
Psychometric Paradigm Factors	Risk perception	- The danger from coronavirus will be fatal to me. - Coronavirus is a serious threat to me and my family.	0.859
	Benefit perception	- If the coronavirus problem is solved, it will greatly benefit our society. - Once the coronavirus is resolved, our society will develop greatly.	0.812
	Trust in government	- The government has the capacity to control the spread of the coronavirus. - The government has a well-prepared preventive system in place for the coronavirus problem.	0.861
	Trust in experts	How much trust do you have in information on the coronavirus from the following organizations and people? - the World Health Organization - doctors → Response scale: (1) extremely distrust; (2) slightly distrust; (3) usually trust; (4) slightly trust; (5) extremely trust.	0.448
	Trust in science	- Thanks to science and technology, the earth’s resources will not be depleted but will become abundant. - Science and technology solve many social problems rather than causing them.	0.754
	Negative affect	- When it comes to coronavirus, negative feelings come first. - Negative images immediately come to mind when I think of coronavirus.	0.910
	Knowledge	- I have good knowledge about the COVID-19 pandemic. - I know more about COVID-19 than others do.	0.840

**Table 3:** Frequency of beliefs in conspiracy theories.

		Preventive Actions		Vaccination Intentions		Beliefs in Conspiracies	
		Mean	*p*-Value	Mean	*p*-Value	Mean	*p*-Value
All respondents		3.771	-	2.736		2.691	
Gender	Male	3.667	0.000	2.758	0.290	2.704	0.516
	Female	3.867		2.715		2.680	
Age	18–29	3.643	0.000	2.646	0.014	2.704	0.596
	30–39	3.745		2.702		2.712	
	40–49	3.712		2.749		2.720	
	50–59	3.810		2.685		2.631	
	60+	3.879		2.839		2.696	
Household income	<299 MW	3.756	0.195	2.737	0.257	2.762	0.035
	300–499 MW	3.753		2.699		2.658	
	>500 MW	3.810		2.781		2.656	
Education level	High school	3.749	0.145	2.797	0.004	2.711	0.337
	College	3.790		2.681		2.674	
No. of children	0	3.772	0.845	2.712	0.130	2.652	0.004
	1	3.756		2.763		2.778	
	2+	3.786		2.829		2.800	
No. of elderly people	0	3.718	0.000	2.697	0.056	2.679	0.724
	1	3.841		2.751		2.699	
	2+	3.838		2.811		2.714	
Ideology	Conservative	3.753	0.226	2.744	0.686	2.816	0.000
	Progressive	3.787		2.728		2.581	
Health status change after COVID-19	Not worse	3.777	0.719	2.554	0.000	2.459	0.000
	Worse	3.767		2.877		2.872	

Note: The mean scores range from 1 to 5.

**Table 4:** Simple and partical correlations among health belief and psychometric factors.

		1	2	3	4	5	6	7	8	9	10	11	12	13	14	15	16	17
1. Preventive actions			0.105 ***	−0.013	−0.078 ***	0.192 ***	−0.069 ***	0.195 ***	0.368 ***	0.140 ***	−0.017	0.243 ***	0.312 ***	0.233 ***	0.150 ***	0.131 ***	0.174 ***	0.240 ***
2. Vaccination intentions		0.121 ***		0.124 ***	0.172 ***	0.074 ***	0.167 ***	0.047 *	−0.054 **	0.102 ***	0.012	0.164 ***	0.061 **	0.062 **	0.120 ***	0.163 ***	0.025	0.155 ***
3. Beliefs in conspiracy theories		−0.008	0.183 ***		0.096 ***	−0.066 **	0.257 ***	−0.094 ***	−0.182 ***	0.054 *	0.023	0.050 *	−0.167 ***	−0.253 ***	−0.014	0.048 *	−0.066 **	−0.069 ***
Health Belief Factors	4. Perceived susceptibility	−0.056 *	0.223 ***	0.159 ***		0.208 ***	0.159 ***	0.050 *	−0.104 ***	−0.024	0.054 **	0.152 ***	−0.075 **	−0.034	−0.046 *	−0.048 *	0.079 ***	0.079 ***
	5. Perceived severity	0.227 ***	0.110 ***	−0.028	0.237 ***		−0.048 *	0.165 ***	0.256 ***	0.038	−0.028	0.458 ***	0.192 ***	0.024	−0.068 ***	−0.033	0.592 ***	0.164 ***
	6. Perceived barriers	−0.064 *	0.223 ***	0.317 ***	0.224 ***	−0.008		0.028	−0.129	0.036	0.009	0.029	−0.107	−0.098	0.031	0.100 ***	−0.053	0.014
	7. Perceived benefits	0.196 ***	0.035	−0.120 ***	0.045	0.164 ***	0.017		0.307 ***	0.054	0.001	0.102 ***	0.195 ***	0.224 ***	0.054	0.121 ***	0.174 ***	0.187 ***
	8. Self−efficacy	0.364 ***	−0.090 ***	−0.239 ***	−141 ***	0.236 ***	−0.175 ***	0.321 ***		0.086	−0.026	0.179 ***	0.310	0.240	0.067	0.104	0.278 ***	0.140
	9. Media exposure	0.154 ***	0.106 ***	0.063 *	−0.007	0.056 *	0.046	0.057 *	0.083 **		0.033	0.090 ***	0.096 ***	−0.019	0.337 ***	0.141 ***	0.031	0.107 ***
	10. Knowing a confirmed case	−0.018	0.011	0.019	0.052 *	−0.032	0.009	0.004	−0.025	0.037		−0.018	−0.011	−0.007	−0.010	−0.011	−0.032	0.045 *
Psychometric Factors	11. Risk perception	0.266 ***	0.218 ***	0.109 ***	0.210 ***	0.487 ***	0.096 ***	0.096 ***	0.134 ***	0.107 ***	−0.018		0.251 ***	0.074 *	0.039	0.012	0.350 ***	0.120 ***
	12. Benefit perception	0.309 ***	0.031	−0.214 ***	−0.094 ***	0.177 ***	−0.134 ***	0.219 ***	0.337 ***	0.101 ***	−0.002	0.221 ***		0.348 ***	0.091 ***	0.156 ***	0.177 ***	0.173 ***
	13. Trust in government	0.220 ***	0.020	−0.320 ***	−0.060 *	0.024	−0.133 ***	0.251 ***	0.280 ***	−0.012	−0.001	0.051 *	0.390 ***		0.109 ***	0.137 ***	0.024	0.134 ***
	14. Trust in experts	0.171 ***	0.121 ***	−0.022	−0.045	−0.050	0.022	0.055 *	0.077 **	0.335 ***	−0.012	0.049	0.094 ***	0.108 ***		0.198 ***	−0.034	0.069 ***
	15. Trust in science	0.133 ***	0.170 ***	0.05	−0.034	−0.034	0.107 ***	0.126 ***	0.099 ***	0.131 ***	−0.008	0.017	0.155 ***	0.117 ***	0.196 ***		0.001	0.141 ***
	16. Negative affect	0.194 ***	0.041	−0.031	0.099 ***	0.595 ***	−0.029	0.167 ***	0.256 ***	0.057 *	−0.030	0.364 ***	0.169 ***	0.012	−0.027	−0.008		0.128 ***
	17. Knowledge	0.254 ***	0.169 ***	−0.065 *	0.103 ***	0.172 ***	0.035	0.205 ***	0.142 ***	0.114 ***	0.052 *	0.143 ***	0.198 ***	0.155 ***	0.070 **	0.162 ***	0.128 ***	

Note: * *p* < 0.05; ** *p* < 0.01; *** *p* < 0.001.; On the diagonal, the lower part is the simple correlation, and the upper part is the partial correlation; In partial correlations, we controlled gender (female), age, household income, education level, no. of children, no. of elderly people, ideology (progressive), health status change after COVID-19; Numbers in simple correlation ranged from 1523 to 1525.

**Table 5:** Multiple regression analysis for preventive action.

		Model 1			Model 2			Model 3			Model 4		
		B	SE	Beta	B	SE	Beta	B	SE	Beta	B	SE	Beta
F1: Sociodemographic Factors	Constant	2.821	0.105		1.401	0.158		1.087	0.138		0.779	0.162	
	Gender (female)	0.200 ***	0.027	0.183	0.155 ***	0.025	0.142	0.195 ****	0.025	0.178	0.174 ***	0.024	0.159
	Age	0.006 ***	0.001	0.153	0.004 ***	0.001	0.099	0.004 ***	0.001	0.113	0.003 **	0.001	0.086
	Household income	0.058 *	0.030	0.049	0.043	0.028	0.036	0.024	0.028	0.020	0.021	0.026	0.017
	Education level	0.109 ***	0.029	0.100	0.073 ***	0.027	0.066	0.060 **	0.027	0.055	0.520 *	0.026	0.047
	No. of children	0.047	0.032	0.039	0.048	0.029	0.040	0.035	0.029	0.029	0.037	0.028	0.031
	No. of elderly people	0.097 ***	0.033	0.088	0.128 ***	0.030	0.116	0.080 ***	0.030	0.073	0.100 **	0.029	0.091
	Ideology (progressive)	0.025 ***	0.008	0.082	0.014 **	0.007	0.047	−0.006	0.007	−0.019	−0.005	0.007	−0.017
	Health status change after COVID-19	0.047 ***	0.017	0.073	0.070 ***	0.016	0.108	0.022	0.016	0.034	0.057 ***	0.016	0.087
F2: Health Belief Factors	Perceived susceptibility				−0.048 ***	0.016	−0.072				0.052 **	0.016	−0.078
	Perceived severity				0.077 ***	0.017	0.112				0.030	0.021	0.044
	Perceived barriers				−0.028	0.019	−0.037				−0.030 *	0.018	−0.039
	Perceived benefit				0.065 ***	0.018	0.086				0.025	0.018	0.033
	Self-efficacy				0.228 ***	0.020	0.300				0.175 ***	0.019	0.230
	Media exposure				0.069 ***	0.016	0.098				0.031 *	0.016	0.044
	Knowing a confirmed case				−0.019	0.072	−0.006				−0.030	0.068	−0.009
F3: Psychometric Factors	Risk perception							0.081 ***	0.016	0.132	0.074 ***	0.016	0.120
	Benefit perception							0.121 ***	0.017	0.182	0.081 ***	0.017	0.122
	Trust in government							0.086 ***	0.016	0.138	0.067 ***	0.016	0.107
	Trust in experts							0.062 ***	0.015	0.094	0.049 **	0.015	0.094
	Trust in science							0.027	0.017	0.037	0.016	0.017	0.022
	Negative affect							0.050 ***	0.016	0.076	0.002	0.018	0.003
	Knowledge							0.129 ***	0.020	0.154	0.119 ***	0.019	0.142
Beliefs in conspiracy theories		−0.009	0.019	−0.012	0.052 ***	0.019	0.071	0.047 **	0.018	0.063	0.077 ***	0.018	0.104
F-value		15.300 ***			30.065 ***			32.696 ***			30.586 ***		
R^2^/Adjusted R^2^		0.083/0.078			0.242/0.234			0.258/0.250			0.320/0.309		

Note: * *p* < 0.05; ** *p* < 0.01; *** *p* < 0.001.

**Table 6:** Multiple regression analysis for vaccination intentions.

		Model 1			Model 2			Model 3			Model 4		
		B	SE	Beta	B	SE	Beta	B	SE	Beta	B	SE	Beta
F1: Sociodemographic Factors	Constant	1.728	0.151		0.908	0.242		0.481	0.212		0.212	0.255	
	Gender (female)	−0.064 *	0.039	−0.041	−0.051	0.038	−0.033	−0.047	0.038	−0.030	−0.023	0.038	−0.014
	Age	0.003 *	0.002	0.056	0.003 *	0.002	0.052	0.001	0.002	0.026	0.002	0.002	0.035
	Household income	0.110 **	0.043	0.064	0.097 **	0.043	0.056	0.068	0.042	0.040	0.063	0.042	0.036
	Education level	−0.094 **	0.042	−0.060	−0.092 **	0.041	−0.059	−0.118 ***	0.041	−0.075	−0.108 ***	0.040	−0.069
	No. of children	0.087 **	0.046	0.051	0.098 **	0.045	0.057	0.085 *	0.044	0.049	0.092 **	0.044	0.053
	No. of elderly people	0.061 *	0.047	0.038	0.077 *	0.046	0.049	0.026	0.046	0.017	0.033	0.046	0.021
	Ideology (progressive)	0.013	0.011	0.030	0.006	0.011	0.014	−0.006	0.011	−0.013	−0.010	0.011	−0.023
	Health status change after COVID-19	0.175 ***	0.024	0.188	0.104 ***	0.025	0.111	0.134 ***	0.024	0.144	0.082 ***	0.025	0.088
F2: Health Belief Factors	Perceived susceptibility				0.122 ***	0.025	0.129				0.116 ***	0.025	0.122
	Perceived severity				0.059 **	0.026	0.059				0.025	0.032	0.026
	Perceived barriers				0.132 ***	0.029	0.121				0.113 ***	0.028	0.104
	Perceived benefit				0.047 *	0.028	0.043				0.004	0.028	0.003
	Self-efficacy				−0.053 *	0.030	−0.049				−0.102 ***	0.030	−0.093
	Media exposure				0.095 ***	0.025	0.094				0.040	0.026	0.039
	Knowing a confirmed case				0.000	0.110	0.000				−0.004	0.107	−0.001
F3: Psychometric Factors	Risk perception							0.131 ***	0.024	0.149	0.106 ***	0.025	0.120
	Benefit perception							−0.012	0.026	−0.013	0.022	0.026	0.023
	Trust in government							0.043 *	0.025	0.048	0.059 **	0.025	0.066
	Trust in experts							0.072 ***	0.023	0.077	0.067 ***	0.024	0.071
	Trust in science							0.121 ***	0.026	0.116	0.119 ***	0.026	0.113
	Negative affect							−0.028	0.025	−0.030	−0.022	0.029	−0.023
	Knowledge							0.148 ***	0.030	0.123	0.130 ***	0.030	0.107
Beliefs in conspiracy theories		0.136 ***	0.028	0.128	0.082 ***	0.029	0.077	0.141 ***	0.028	0.133	0.092 ***	0.029	0.087
F-value		15.265			14.636			16.842			15.128 ***		
R^2^/Adjusted R^2^		0.083/0.078			0.135/0.125			0.152/0.143			0.189/0.176		

Note: * *p* < 0.05; ** *p* < 0.01; *** *p* < 0.001.

## Data Availability

The data presented in this study are available on request from the corresponding author. The data are not publicly available due to regulations and guideline of data open policy according to the National Research Foundation of Korea.

## Appendix A

**Table A1:** Dependent Variables: Preventive Action.

	B	SE	Beta	B	SE	Beta
Benefit perception	0.081 ***	0.017	0.122	0.084 ***	0.017	0.127
Conspiracy	0.077 ***	0.018	0.104	0.082 ***	0.018	0.111
Interaction term	−			−0.046 *	0.018	−0.055
F-value	30.586 ***			29.683 ***		
R^2^ square	0.320			0.323		
R^2^ square change	0.309			0.312		
Simple slope test	Law	B = 0.118 *** se = 0.022 t = 5.306				
	Middle	B = 0.084 *** se = 0.017 t = 5.008				
	High	B = 0.050 ** se = 0.021 t = 2.421				
Effect size	0.005					
	B	SE	beta	B	SE	beta
Trust in government	0.067 ***	0.016	0.107	0.077 ***	0.016	0.123
Conspiracy	0.077 ***	0.018	0.104	0.084 ***	0.018	0.114
Interaction term	-			−0.048 **	0.015	−0.069
F-value	30.586 ***			29.878 ***		
R^2^ square	0.320			0.324		
R^2^ square change	0.309			0.313		
Simple slope test	Law	B = 0.112 *** se = 0.022 t = 5.186				
	Middle	B = 0.077 *** se = 0.016 t = 4.719				
	High	B = 0.042 ** se = 0.018 t = 2.327				
Effect size	0.006					

Note: * *p* < 0.05; ** *p* < 0.01; *** *p* < 0.001.

**Table A2:** Dependent Variable: Vaccination.

	B	SE	Beta	B	SE	Beta
Trust in government	0.059 *	0.025	0.066	0.041	0.026	0.046
Conspiracy	0.092 **	0.029	0.087	0.079 **	0.029	0.075
Interaction term	-			0.089 ***	0.024	0.091
F-value	15.128 ***			15.194 ***		
R² square	0.189			0.199		
R² square change	0.176			0.183		
Simple slope test	Law	B = −0.025 se = 0.034 t = −0.739				
	Middle	B = 0.041 se = 0.026 t = 1.605				
	High	B = 0.107 *** se = 0.028 t = 3.803				
Effect size	0.040					
	B	SE	beta	B	SE	beta
Trust in science	0.119 ***	0.026	0.113	0.121 ***	0.026	0.115
Conspiracy	0.092 **	0.029	0.087	0.085 **	0.029	0.08
Interaction term	-			0.08 **	0.029	0.066
F-value	15.128 ***			14.877 ***		
R² square	0.189			0.193		
R² square change	0.176			0.18		
Simple slope test	Law	B = 0.062 se = 0.033 t = 1.853				
	Middle	B = 0.121 *** se = 0.026 t = 4.615				
	High	B = 0.179 *** se = 0.034 t = 5.248				
Effect size	0.016					

Note: * *p* < 0.05; ** *p* < 0.01; *** *p* < 0.001.

## Appendix B

**Table A3:** Simple Correlation between Preventive Action/Vaccination and Seven Measures for Belief in Conspiracy Theories.

	1	2	3	4	5	6	7	8
1. Preventive action	1							
2. Vaccination	0.121 ***	1						
3. The government makes important decisions related to coronavirus disease (COVID-19) without the public knowing.	0.014	0.172 ***	1					
4. Politicians do not honestly reveal their true intentions to the public regarding their decisions on coronavirus disease (COVID-19).	0.049 *	0.011	0.344 ***	1				
5. The government is hiding something from the public.	−0.007	0.156 ***	0.611 ***	0.384 ***	1			
6. There is a secret organization that greatly influences political decisions.	−0.004	0.115 ***	0.480 ***	0.377 ***	0.682 ***	1		
7. The government is always monitoring the public.	0.004	0.175 ***	0.544 ***	0.351 ***	0.665 ***	0.642 ***	1	
8. Certain powerful nations deliberately created the coronavirus (COVID-19) to dominate the world.	−0.026	0.146 ***	0.389 ***	0.204 ***	0.416 ***	0.455 ***	0.508 ***	1
9. Coronavirus disease (COVID-19) was deliberately created by pharmaceutical companies to make money.	−0.069 ***	0.152 ***	0.356 ***	0.092 ***	0.355 ***	0.397 ***	0.408 ***	0.721 ***

Note: * *p* < 0.05; *** *p* < 0.001.
